# Discovery of monoclonal antibodies cross-reactive to novel subserotypes of *K. pneumoniae* O3

**DOI:** 10.1038/s41598-017-06682-2

**Published:** 2017-07-26

**Authors:** Luis M. Guachalla, Katarina Stojkovic, Katharina Hartl, Marta Kaszowska, Yadhu Kumar, Benjamin Wahl, Tobias Paprotka, Eszter Nagy, Jolanta Lukasiewicz, Gábor Nagy, Valéria Szijártó

**Affiliations:** 1 Arsanis Biosciences GmbH, Helmut Qualtinger-Gasse-2, 1030 Vienna, Austria; 2 Laboratory of Microbial Immunochemistry and Vaccines, Department of Immunochemistry, Ludwik Hirszfeld Institute of Immunology and Experimental Therapy, Polish Academy of Sciences, Rudolfa Weigla 12, 53-114 Wroclaw, Poland; 3 GATC Biotech AG, Jakob-Stadler-Platz 7, 78467 Konstanz, Germany

## Abstract

*Klebsiella pneumoniae* is responsible for nosocomial infections causing significant morbidity and mortality. Treatment of newly emerging multi-drug resistant strains is hampered due to severely limited antibiotic choices. Passive immunization targeting LPS O-antigens has been proposed as an alternative therapeutic option, given the limited variability of Klebsiella O-antigens. Here we report that the O3 serogroup, previously considered to have uniform O-antigen built of mannan, represents three different subtypes differing in the number of mannose residues within the O-antigen repeating units. Genetic analysis of the genes encoding mannose polymerization revealed differences that underline the observed structural alterations. The O3 variants represent antigenically different types based on the different reactivity pattern of murine monoclonal antibodies raised against a *K. pneumoniae* O3 strain. Typing of a collection of *K. pneumoniae* O3 clinical isolates showed that strains expressing the novel O3b antigen, the tri-mannose form, were more prevalent than those having the penta-mannose form, traditionally called O3, while the tetra-mannose variant, termed here O3a, seems to be rare. A monoclonal antibody cross-reacting with all three O3 sub-serogroups was also selected and shown to bind to the surface of various *K. pneumoniae* strains expressing different O3 subtypes and capsular antigens.

## Introduction

Carbapenem resistant *Enterobacteriaceae* (CRE) are recognized as urgent threat level pathogens requiring immediate actions (CDC: Antibiotic Resistance Threats, 2013). Among CRE, *Klebsiella pneumoniae* strains are by far the most common isolates. With acquisition of the recently described plasmid mediated colistin resistance^1^, pan-resistant strains have emerged^2^. Given the dwindling development pipeline of novel antibiotics with Gram-negative coverage, alternative therapeutic options against extremely drug resistant Gram-negative bacteria are needed. Passive immunization using pathogen-specific monoclonal antibodies (mAbs) is an approach envisioned to be in good accordance with proposed antibiotic stewardship efforts. Application of mAbs does not induce antibiotic resistance, preserves the normal microbiota, and hence may also be used for prophylaxis in high-risk patient populations.

*K. pneumoniae* LPS O-antigens show limited variability and consequently were proposed as viable targets for immunization^3–5^. Former and recent epidemiology data suggest that 3 to 4 serogroups constitute more than 80% of all *K. pneumoniae* clinical isolates^4–6^. Efficacy of mAbs against strains expressing the most common galactan-based O-specific polysaccharides (O-PS) (O1 and O2) was previously shown^7, 8^. The remaining common serogroups, O3 and O5, express O-PS chains made up of mannan (i.e. homopolymer of mannose) structures that are identical to the *E. coli* O9 and O8 O-PS structures, respectively^9, 10^. The O3 and O5 repeating units (RU) differ in their number of mannoses, anomeric configurations and/or their intra-mannose linkages^11^. The non-reducing ends of both O3 and O5 side-chains are terminated with different structures, methylphosphate (MeP) and methyl (Me) groups, respectively^11, 12^. O-PS termination is coupled to chain polymerization in the WbdA-WbdD complex, which is anchored in the inner face of the inner membrane^13, 14^. WbdA is a multi-domain mannosyltransferase^15^; different domains of this enzyme catalyse the addition of mannose molecules at different linkages to the O-antigen polymer. A single point mutation in one of the mannosyltransferase domains of WbdA in *E. coli* O9 results in the reduction of 5 mannose molecules to 4 within the RU^16^. The WbdD enzymes are responsible for the serogroup-specific termination^17^. O-PS termination is the signal for ABC-mediated transport across the inner membrane^18^ followed by the ligation of O-antigens to the preformed lipid A-core templates.

In this study, we describe novel *K. pneumoniae* O3 subtypes that express O-antigen RUs comprising 3 or 4 mannoses in contrast to the 5 mannoses originally described. The novel variants carry mutations in the mannosyltransferase domains of WbdA, which can explain the observed structural differences. Moreover, in support of the clinical significance, we have selected two classes of mAbs that are either specific to certain subtypes or cross-reactive to all O3 variants and therefore can be considered for diagnostic (seroepidemiology) or therapeutic purposes, respectively.

## Results

### Development of mAbs with different specificity to O3 strains

**Figure 1:**
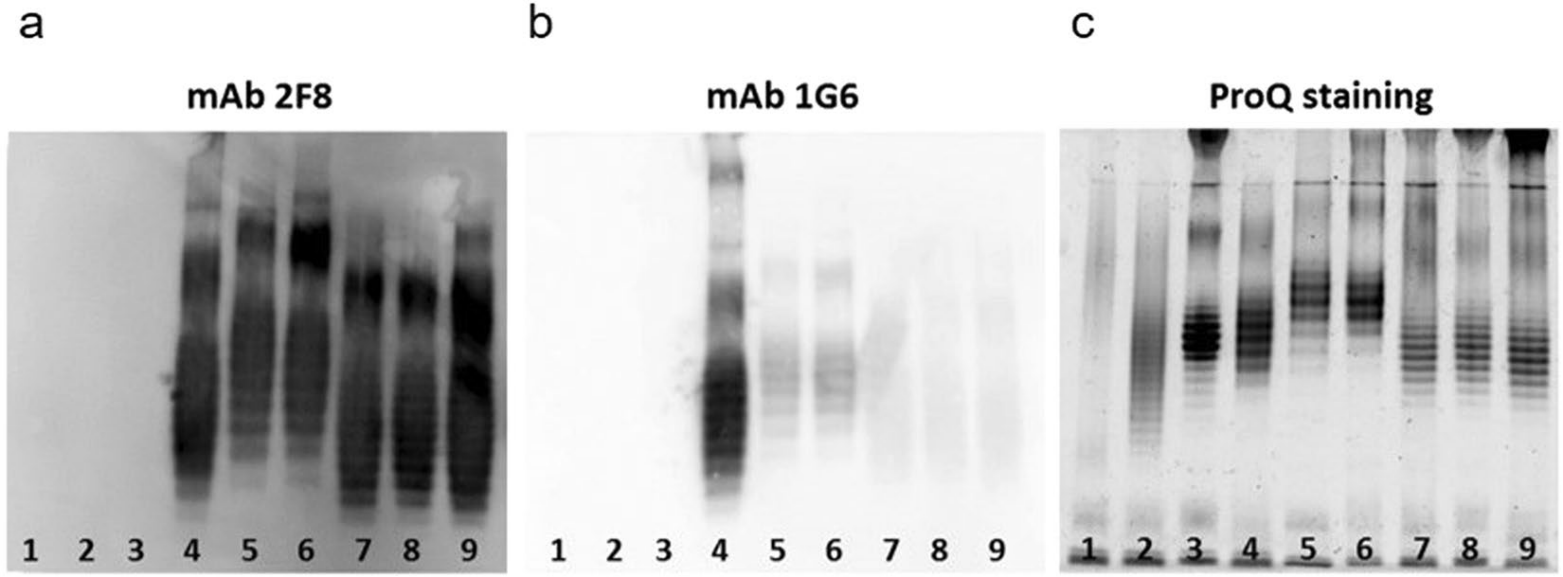
Immunoblot (**a**,**b**) and ProQ staining (**c**) of LPS samples. LPS was purified from different O3 (lanes 4–9) and unrelated serotype (lanes 1–3) strains of *K. pneumoniae*. Immunoblots were performed with 1 μg/ml of mAbs 2F8 (**a**) or 1G6 (**b**). Lane 1: ATCC43816 (O1:K2), 2: PCM-27 (O2:K27), 3: Kp108 (O5), 4: PCM-11, 5: Kp14, 6: Kp62, 7: Kp18, 8: Kp35, 9: Kp82.

Mouse hybridomas were generated using splenocytes from BALB/c mice immunized with *K. pneumoniae* prototype strain PCM-11 (O3:K11: this strain, in fact, is proven in this study to express an O3a type O-antigen). Reactive clones specific to the purified and biotinylated O-PS of a non-encapsulated mutant, *K. pneumoniae* 5505Δ*cps* (O3:K-) were selected by screening hybridoma supernatants by ELISA. Subsequently, the purified mAbs were tested by immunoblotting with a panel of LPS samples obtained from different *K. pneumoniae* O3 clinical isolates identified by O3-specific PCR using primers specific to the *wzm* genes (Fig. 1). Interestingly, two classes of mAbs were found: i) those reacting with LPS from all O3 strains (exemplified by mAb 2F8, Fig. 1a) and ii) those showing different binding intensities (from strong to negligible binding) to LPS from various O3 strains (as shown with mAb 1G6, Fig. 1b). None of these mAbs stained any of the non-O3 LPS molecules, such as O1, O2, and O5 (Fig. 1, lanes 1, 2, and 3, respectively) indicating lack of poly-reactivity.

**Figure 2:**
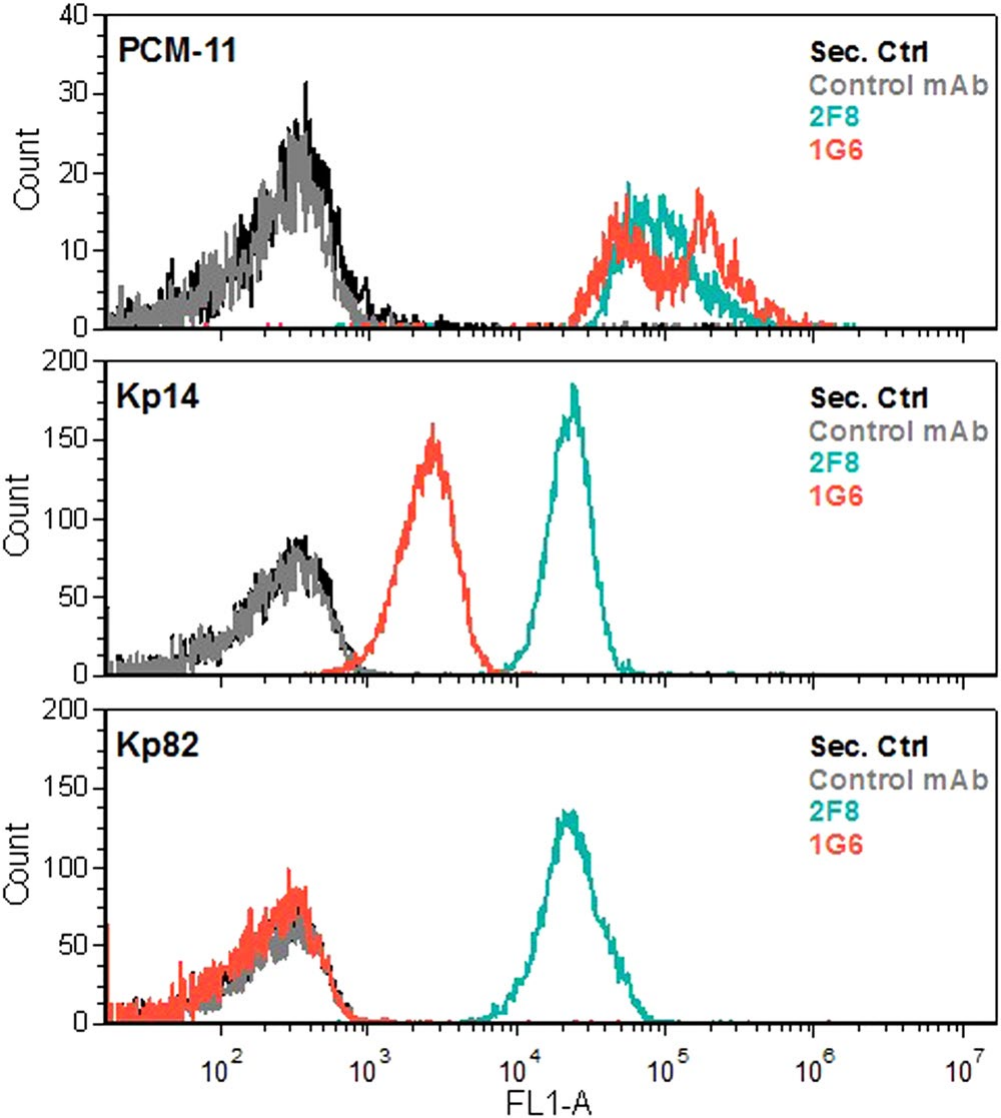
Surface staining of live *K. pneumoniae* O3 strains with monoclonal antibodies. O3-specific or a control murine mAb was incubated with live Klebsiella cells of the indicated strains. The binding was detected with a labelled anti-mouse IgG by flow cytometry.

Three O3 strains exhibiting different binding to mAb 1G6 (Fig. 1b), i.e. PCM-11 (the immunizing strain), Kp14 (showing a weak but detectable binding) and Kp82 (with negligible binding) were selected for flow cytometry analysis in order to investigate mAb binding to native antigens on the surface of live bacteria (Fig. 2). The immunizing strain was stained comparably strong by mAbs 1G6 and 2F8. In contrast, strain Kp14 was stained much weaker, while strain Kp82 was not bound at all by 1G6. All O3 strains showed comparably strong surface binding to the cross-reactive mAb 2F8. These data correlated well with the immunoblotting results (Fig. 1). To confirm the surface staining pattern observed with the two mAbs, a larger panel of O3 clinical isolates identified by PCR were tested by surface staining (Suppl. Fig. 1). While all strains were stained by mAb 2F8 independent of their capsule type, mAb 1G6 showed binding only to a subset of clinical isolates. The concordant flow cytometry (Fig. 2) and immunoblot data (Fig. 1a,b), together with the different staining pattern observed for the separated LPS molecules (Fig. 1c), suggested that structural differences among the O3 O-PSs could be responsible for the different binding to the various antibodies. However, the different types were likely to share common epitopes as well, which was corroborated by the selection of the cross-reactive mAb (2F8).

### Structural analysis of various O3 subtypes

O-specific polysaccharides (O-PS) isolated from three strains (PCM-11, Kp81 and 5505Δ*cps*), representing the putative different structural variants, were analysed by chemical methods (sugar and methylation analyses), NMR spectroscopy, and MALDI-TOF mass spectrometry (MS). Combined results of sugar and methylation analyses (data not shown) confirmed the presence of 2- and 3-substituted mannopyranoses (Man*p*).

**Figure 3:**
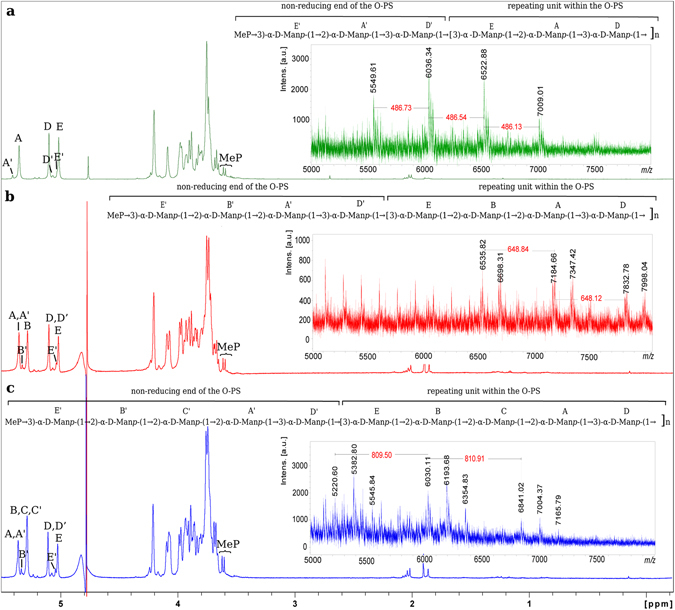
^1^H-NMR spectra and structures of O-specific polysaccharides isolated from *K. pneumoniae* Kp81 (**a**), PCM-11 (**b**), and 5505Δ*cps* (**c**). The 1H-NMR spectrum was obtained for a D_2_O solution at 600 MHz (25 °C). The Arabic numerals refer to protons in the respective residues. Letters with a prime sign denote residues of terminal units of each O-PS. O-PS RUs are marked off by brackets. Methyl phosphate (MeP) group represents modification at the non-reducing end of the O-specific polysaccharide. The negative-ion mode MALDI-TOF mass spectra of O-PSs showing mass differences between O-PS ions indicating tri-, tetra-, and pentasaccharide RU of Kp81, PCM-11 and 5505Δ*cps*, respectively (right panels).

The average molecular weights of the O-PS RUs were determined by negative ion mode MALDI-TOF MS (Fig. 3). Mass spectra revealed clusters of ions attributed to different lengths of O-PS with average monoisotopic mass differences of 486.5, 648.5 and 810.2 Da matching the calculated monoisotopic mass of RUs composed of 3 (486.16 Da), 4 (648.21), or 5 (810.26) Man, respectively. Due to the high laser power level used to improve polysaccharide ionisation, all mass spectra showed partial in-source degradation of the O-PS reflected by ions with mass differences of approx. 162 Da attributing to the loss of Man within the lower *m/z* region (Fig. 3b and data not shown for 3a and 3c). In-source degradation resulted in observation of the set of ions (e.g. *m/z* 6535.82 and *m/z* 6698.31; *m/z* 7184.66 and *m/z* 7347.42) differing by 162 Da (Fig. 3b).

The complete assignments of ^1^H and ^13^C resonances for all three O-PSs were performed by interpretation of one- and two-dimensional NMR data (Table 1), including COSY, TOCSY, NOESY, HMBC, HSQC-DEPT, and HSQC-TOCSY. The 1D ^1^H NMR (Fig. 3) and ^1^H, ^13^C HSQC-DEPT spectra (data not shown) obtained for Kp81, PCM-11, and 5505Δ*cps* O-PSs contained signals for three (residues A, D, E), four (residues A, B, D, E), or five (residues A, B, C, D, E) major anomeric protons and carbons of α-d-Man*p*, respectively (Fig. 3, Table 1). Chemical shift values for the identified Man*p* were compared to respective residues reported previously for the pentasaccharide RU of *K. pneumoniae* O3^11, 12^. Additionally, methylphosphate (MeP) was identified on the basis of the presence of two sharp proton signals of methyl group (for Kp81: δ_H_ 3.61, 3.63; δ_C_ 53.7 ppm; for PCM-11: δ_H_ 3.61, 3.63; δ_C_ 53.9 ppm; for 5505Δ*cps*: δ_H_ 3.61, 3.63; δ_C_ 53.8 ppm) as a doublet with *J*_P,H_ of 11.0 Hz indicating its substitution by phosphate group (MeP). MeP group represents known modification at the non-reducing end of the O3 O-PS and serves as the signal for termination of the O-PS elongation^12^.

**Table 1:** ^1^H and ^13^C NMR chemical shifts and selected inter-residue NOE and ^3^ *J*_H,C_ connectivities from O-PS isolated from *K. pneumoniae* Kp81, PCM-11, and 5505Δ*cps*.

Residue	Strain	Chemical shifts (ppm)						Selected inter-residue NOE and ^3^ *J*_H,C_ connectivities	
		H1/C1	H2/C2	H3/C3	H4/C4	H5/C5	H6/C6	H1/C1 connectivities to	Inter-residue atom/residue
A → 2)-α-d-Man*p*-(1→	Kp81	5.36/101.4	4.10/79.0	3.98/70.8	3.69/67.8	3.77/74.1	3.76,3.90/61.7	3.99/79.0	H3, C3 of D
	PCM-11	5.37/101.5	4.10/79.5	4.00/70.9	3.69/67.9	3.77/74.2	3.76,3.92/61.9	4.00/79.2	H3, C3 of D
	5505	5.37/101.4	4.07/79.5	3.98/70.8	3.68/67.7	3.78/74.1	3.74,3.89/61.8	3.98/79.2	H3, C3 of D
A’ → 2)-α-d-Man*p*-(1→	Kp81	5.42/101.2	4.09/79.8	3.96/70.8	3.72/67.8	3.77/74.1	3.76, 3.90/61.7	4.01/79.0	H3, C3 of D’
	PCM-11	5.37/101.5	4.10/79.5	4.00/70.9	3.69/67.9	3.77/74.2	3.76,3.92/61.9	4.00/79.2	H3, C3 of D’
	5505	5.37/101.4	4.07/79.5	3.98/70.8	3.68/67.7	3.78/74.1	3.74,3.89/61.8	3.98/79.2	H3, C3 of D’
B → 2)-α-d-Man*p*-(1→	Kp81	na	na	na	na	na	na	na	na
	PCM-11	5.30/101.5	4.11/79.4	3.97/70.9	3.73/67.7	3.76/74.1	3.76, 3.86/61.8	4.10/79.5	H2, C2 of A
	5505	5.29/101.4	4.10/79.3	3.94/70.8	3.69/67.8	3.74/74.1	3.74, 3.84/61.8	4.08/79.5	H2, C2 of C
B’ → 2)-α-d-Man*p*-(1→	Kp81	na	na	na	na	na	na	na	na
	PCM-11	5.35/101.4	4.11/80.1	3.93/70.8	3.72/67.7	3.74/74.2	3.76,3.86/61.8	4.10/79.6	H2, C2 of A’
	5505	5.34/101.3	4.09/80.0	3.91/70.8	3.72^b^/67.7	3.74^b^/74.0	3.75^b^, 3.84/61.8	4.07/79.6	H2, C2 of C’
C → 2)-α-d-Man*p*-(1→	Kp81	na	na	na	na	na	na	na	na
	PCM-11	na	na	na	na	na	na	na	na
	5505	5.29/101.4	4.08/79.5	3.94/70.8	3.69/67.8	3.73/74.1	3.74,3.84/61.8	4.07/79.5	H2, C2 of A
C’ → 2)-α-d-Man*p*-(1→	Kp81	na	na	na	na	na	na	na	na
	PCM-11	na	na	na	na	na	na	na	na
	5505	5.29/101.4	4.08/79.5	3.94/70.8	3.69/67.8	3.73/74.1	3.74,3.84/61.8	4.07/79.5	H2, C2 of A’
D → 3)-α-d-Man*p*-(1→	Kp81	5.12/102.7	4.22/70.3	3.99/79.0	3.77/66.8	3.81/74.2	3.76, 3.91/61.7	3.94/78.7	H3, C3 of E
	PCM-11	5.12/102.9	4.24/70.5	4.00/79.2	3.75/67.0	3.82/74.4	3.76, 3.92/61.9	3.94/78.9	H3, C3 of E
	5505	5.11/102.9	4.21/70.4	3.98/79.2	3.75/66.9	3.81/74.2	3.73, 3.90/61.8	3.93/78.8	H3, C3 of E
D’ → 3)-α-d-Man*p*-(1→	Kp81	5.09/102.8	4.20/70.3	4.01/79.0	3.77/66.8	3.81/74.2	3.76, 3.91/61.7	3.94/78.7	H3, C3 of E
	PCM-11	5.12/102.9	4.24/70.5	4.00/79.2	3.75/67.0	3.82/74.4	3.76, 3.92/61.9	3.94/78.9	H3, C3 of E
	5505	5.11/102.9	4.21/70.4	3.98/79.2	3.75/66.9	3.81/74.2	3.73, 3.90/61.8	3.93/78.8	H3, C3 of E
E → 3)-α-d-Man*p*-(1→	Kp81	5.04/102.8	4.22/70.3	3.94/78.7	3.77/66.8	3.77/74.1	3.76, 3.84/61.6	4.10/79.0	H2, C2 of A
	PCM-11	5.03/102.9	4.23/70.4	3.94/78.9	3.75/67.0	3.80/74.2	3.76, 3.92/61.9	4.11/79.4	H2, C2 of B
	5505	5.03/102.9	4.21/70.4	3.93/78.8	3.75/66.9	3.79/74.2	3.73, 3.88/61.9	4.10/79.3	H2, C2 of B
E’ α-d-Man*p*PMe-(1→	Kp81	5.05/102.8	4.25/69.9	4.26/76.4^a^	3.77/66.3	3.81/74.1	3.76, 3.84/61.6	4.08/79.8	H2, C2 of A’
	PCM-11	5.05/103.0	4.26/70.0	4.26/76.6^a^	3.78/66.9	3.85/74.3	3.74, 3.86/61.8	4.10/80.1	H2, C2 of B’
	5505	5.05/102.8	4.24/70.0	4.25/76.6^a^	3.76/66.5	3.84/74.0	3.75, 3.86/61.8	4.09/80.1	H2, C2 of B’

^a^MeP was identified in Kp81: 3.61, 3.63/53.7 ppm; in PCM-11: 3.61, 3.63/53.9 ppm; and in 5505Δ*cps*: 3.61, 3.63/53.8 ppm, where *J*_P,H_ was 11.0 Hz. ^31^P, ^1^H HMBC showed correlation between P (~2 ppm) and protons of MeP and H-3 of **E’** residues. ^31^P, ^1^H HMQC-TOCSY showed correlation between P and H-1, H-2, H-3, H-4, H-5 of **E’** residues. Anomeric configuration (α) of sugar residues was determined on the basis of *J*_H1,C1_ of 171-174 Hz.

^b^Assignments of resonances were made by comparisons with published data^12^. na- not applicable for indicated strain.

NMR spectra of O-PS of Kp81 indicated the presence of three α-d-Man*p* (residues A, D, E) as constituents of the O-PS RU and minor spin systems of Man*p* (residues A’, D’, E’). Residue A (δ_H_/δ_C_ 5.36/101.4 ppm, ^1^ *J*_C1,H1_ 172 Hz) was recognized as the →2)-α-d-Man*p* due high chemical shift value of C2 (δ_C_ 79.0 ppm). Residue D (δ_H_/δ_C_ 5.12/102.7 ppm, ^1^ *J*_C1,H1_ 171 Hz) was recognized as the →3)-α-d-Man*p* due to the high chemical shift value of C3 (δ_C_ 79.0 ppm). Residue E (δ_H_/δ_C_ 5.04/102.8 ppm, ^1^ *J*_C1,H1_ 172 Hz) was recognized as the →3)-α-d-Man*p* due to the high chemical shift value of C3 (δ_C_ 78.7 ppm). The sequence of sugar residue in the Kp81 RU was confirmed by NOESY and HMBC experiments showing inter-residue connectivities between adjacent residues (Table 1), which confirmed the trisaccharide RU structure →3)-**E**-(1 → 2)-**A**-(1 → 3)-**D**-(1→ (Fig. 3a, inset structure). ^1^H,^31^P HMBC spectrum showed correlation of MeP group to H3 of residue E’ (α-d-Man*p*3PMe). Additionally, ^31^P,^1^H HMQC-TOCSY (data not shown) showed correlation of MeP group to H-2, H-3, H-4, H-5 of residue E’, supporting substitution of E’ by MeP. Furthermore, residues A’ (→2)-α- d-Man*p*) and D’ (→3)-α- d-Man*p*) were identified and together with E’ were found to be constituents of terminal RU at non-reducing end of the Kp81 O-PS. Complete structure of the Kp81 O-PS including biological RU and non-reducing end is presented in Fig. 3a.

Strain PCM-11 was originally deposited at the Polish Collection of Microorganisms (PCM) as a prototype *K. pneumoniae* O3:K11 strain, and it was expected to express the pentamannose RU^11^. However NMR spectra of O-PS of PCM-11 indicated the presence of four α-d-Man*p* (residues A, B, D, E) as constituents of the O-PS RU. Residue A (δ_H_/δ_C_ 5.37/101.5 ppm, ^1^J_C1,H1_ 173 Hz) was recognized as the →2)-α-d-Man*p* due to the high chemical shift value of C2 (δ_C_ 79.5 ppm). Residue B (δ_H_/δ_C_ 5.30/101.5 ppm, ^1^ *J﻿*_C1,H1_ 173 Hz) was recognized as the →2)-α-d-Man*p* due to the high chemical shift value of C2 (δ_C_ 79.4 ppm). Residue D (δ_H_/δ_C_ 5.12/102.9 ppm, ^1^ *J*_C1,H1_ 174 Hz) was recognized as the →3)-α-d-Man*p* due to the high chemical shift value of C3 (δ_C_ 79.2 ppm). Residue E (δ_H_/δ_C_ 5.03/102.9 ppm, ^1^ *J*_C1,H1_ 173 Hz) was recognized as the →3)-α-d-Man*p* due to the high chemical shift value of C3 (δ_C_ 78.9 ppm). The sequence of sugar residues in the PCM-11 RU was confirmed by NOESY and HMBC experiments showing inter-residue connectivities between adjacent residues (Table 1), which confirmed the presence of tetrasaccharide RU structure →3)-**E**-(1 → 2)-**B**-(1 → 2)-**A**-(1 → 3)-**D**-(1→ in *K. pneumoniae* PCM-11 O-PS (Fig. 3b, inset structure). Additionally ^1^H,^31^P HMBC spectrum showed correlation of MeP group to H3 of residue E’ (α-d-Man*p*3PMe). Moreover, ^31^P,^1^H HMQC-TOCSY (data not shown) showed correlation of MeP group to H-2, H-3, H-4, H-5 of residue E’, supporting substitution of E’ by MeP. Identification of residue E’ facilitate identification of minor spin systems of A’, B’, D’ that were constituents of terminal unit of the strain PCM-11 O-PS containing MeP at position 3 of residue E’. Complete structure of the PCM-11 O-PS including biological RU and non-reducing end is presented in Fig. 3b, inset structure.

Analysis of the O-PS isolated from the 5505Δ*cps* strain resulted in the expected O-PS structure built up of five Man*p* residues (Fig. 3c, inset structure)^11, 12^. All previously reported spin systems were identified (residues A, B, C, D, E), including partial identification of minor spin systems (A’, B’, C’, D’, E’) represented by well-resolved signals for residues E’ and B’ attributed to terminal unit of the O-PS containing MeP at position 3 of residue E’. Chemical shift values for the ^1^H and ^13^C resonances were compared with data reported previously^11^.

To differentiate from the original O3 pentamannose structures, the novel tetra- and tri-mannose RU structures have been designated as O3a and O3b, respectively.

### Genetic comparison of different O3 strains

In order to substantiate the structures of the newly identified O3 subtypes, whole genome sequencing of 12 selected O3 strains with an average coverage of at least 170-fold was performed. The sequence reads were assembled into contiguous reads (contigs) and subsequently genes were annotated. The *rfb* (*w* *b*) clusters, encoding O-antigen synthesis and transport, were extracted from assembled contigs for each strain and translated into the corresponding amino acid sequences.

**Figure 4:**
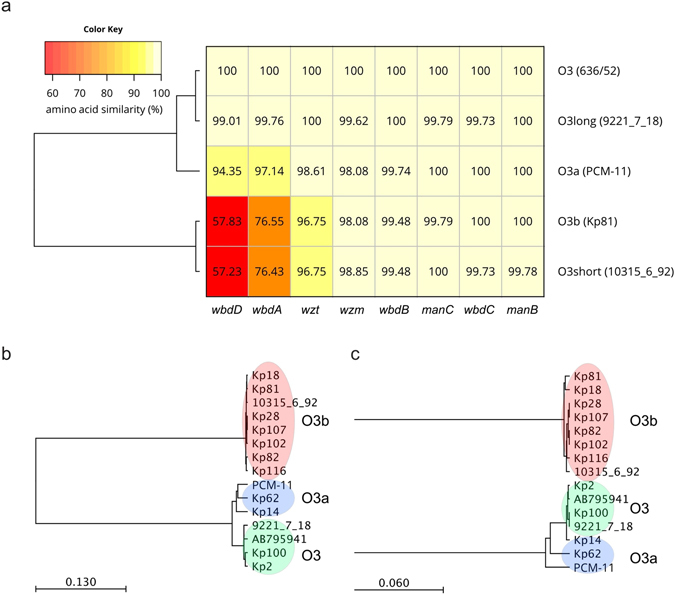
Schematic alignment and taxonomy of the *rfb* operons encoding O3 antigens. The percent homologies of individual gene products compared to those of archetype O3 strain 636/52 (AB795941.1) are shown for representative strains expressing O3, O3a or O3b antigens (**a**). In addition, the two genotypes “O3 short” and “O3 long” of O3 *rfb* operons recently described by Follador *et al*.^5^ are shown. Phylogenetic trees for WbdD (**b**) and WbdA (**c**) were calculated.

Figure 4 shows the draft sequence alignment of each gene product encoded within the *rfb* loci in comparison to the published sequence (accession number: AB795941.1) encoding the archetype penta-mannose O3 RU^11^. Proteins responsible for the synthesis of mannose (ManC and ManB) as well as those responsible for the transport of polymerized O-antigens (Wzm and Wzt) showed high conservation in all variants. Similarly, enzymes encoded at the 3′ end of the operon (WbdB and WbdC) directing the synthesis of the di-saccharide adaptor for O-antigen ligation were well-conserved. In contrast, significantly lower homologies were found in WbdD and WbdA responsible for the synthesis of RUs and the termination of the RU polymers. Although the amino acid sequences of WbdA from O3 and O3a were found relatively similar (97%), the C80R point mutation that had been reported in the analogous *E. coli* O9a isolates, but not in *E. coli* O9^16^, was found in the *K. pneumoniae* O3a WbdA. This amino acid replacement was used as a surrogate for the structural difference between O3 and O3a subunits. Interestingly, the same amino acid replacement was also shown in WbdA of O3b, however, it was accompanied by a significantly higher sequence variability in the entire protein (76.6% homology between O3 and O3b WbdA proteins). The lowest homology was found in the WbdA^N^ mannosyltransferase domain (70.4%) that generates the 1–2 linkage between mannosyl residues (Suppl. Fig. 2). The other mannosyltransferase domain WbdA^C^, responsible for the 1–3 linkage, was relatively well-conserved (87.7% homology), except for the region critical for the interaction between WbdD and WbdA (Val^548^-Ile^568^; ref. 19). In good agreement with this, the overall similarity of the WbdD variants of O3 and O3b was found to be as low as 57.8%. Alignment of the individual domains (Suppl. Fig. 3) of WbdD showed, that compared to the methyltransferase domain (72.6%), the kinase domain shows a lower level of homology (48.7%).

Importantly, when comparing the *rfb/wb* locus of the sequenced clinical isolates expressing O3 or O3b O-antigen, high level of homology was observed among the same serotypes; even alignment of the WbdA and WbdD proteins showed no or minimal differences (Suppl. Figs 2 and 3). When drawing relational dendograms for WbdA and WbdD, strains with the same O3 subtype clustered together (Fig. 4b and c).

Interestingly, the two genotypes of O3 *rfb* operons (i.e. “long” and “short” forms) recently described by Follador *et al*.^5^ matched very well with the O3 and O3b sequences described here (overall nucleotide homology 99.24% and 98.99%).

### Seroepidemiology of O3 subtypes

A total of 31 *K. pneumoniae* O3 isolates deriving from different geographical locations and clinical specimens were genotyped and phenotyped using specific PCRs and reactivity to the newly identified anti-O3 antibodies, respectively (Table 2). As expected, all strains could be identified by pan-O3 specific primers that amplify a serogroup specific gene fragment of *wzm*, which is conserved in all subtypes of the O3 serogroup. Expression of any of the O3 subtype antigens was confirmed by binding to the cross-reactive mAb 2F8. O3b strains were differentiated from the other two serotypes (i.e. O3 and O3a) by serogroup-specific PCR reactions designed based on the sequence divergence of *rfb* loci and by lack of binding by mAb 1G6. Besides the detection of the substantially weaker binding of mAb 1G6 to O3 compared to O3a strain(s) both by immunoblot (data not shown) and by flow cytometry (Suppl. Fig. 1), sequencing of *wbdA* amplicons was also used to differentiate between O3 and O3a based on the polymorphism at amino acid position 80. As shown in Table 2, however, none of the non-O3b clinical isolates carried the C80R mutation, hence the only *K. pneumoniae* strain in this collection belonging to the O3a subtype was strain PCM-11. To rule out the possibility that PCM-11 is indeed not a *K. pneumoniae* isolate, the species was confirmed at the Polish Collection of Microorganisms by MALDI Biotyper® and by biochemical analysis (with 95% likelihood to belong to taxon *K. pneumoniae* subsp*. pneumoniae*).

**Table 2:** Clinical isolates analyzed in the study.

Strain	Country of origin	Resistance profile^a^	MLST	Beta-lactamase	Specimen	PCR			Immunoblot		*wbdA* sequencing	Serotype	Accession number	K-type^c^
						Pan-O3	O3/O3a	O3b	2F8	1G6				
Kp2	Hungary	ESBL	ST1915	nd	urine	+	+	−	+	+	C at position 80	**O3**	SRR5270320	K60
Kp14	Hungary	ESBL	ST113	SHV	central venous route	+	+	−	+	+	C at position 80	**O3**	SRR5270322	K35
Kp18	Hungary	ESBL	ST834	CTX-M	blood culture	+	−	+	+	−		**O3b**	SRR5270321	Non typeable
Kp28	Hungary	ESBL	ST323	SHV	central venous route	+	−	+	+	−		**O3b**	SRR5270319	K51
Kp35	Poland	ESBL, CR	ST11	CTX-M-3, SHV-12, KPC-2	na	+	−	+	+	−		**O3b**	SRR5270318	Non typeable
Kp49	Spain	CR	ST54	VIM-1	urine	+	−	+	+	−		**O3b**		nd
Kp62	Spain	CR	ST384	KPC-3	urine	+	+	−	+	+	C at position 80	**O3**	SRR5270317	K35
Kp72	Spain	ESBL	ST14	TEM-4	na	+	−	+	+	−		**O3b**		nd
Kp77	Spain	ESBL	ST385	SHV-12	urine	+	−	+	+	−		**O3b**	SRR5270316	Non typeable
Kp81	Spain	ESBL	ST346	SHV-12	wound	+	−	+	+	−		**O3b**	SRR5270315	Non typeable
Kp82	Spain	ESBL	ST370	SHV-12	wound	+	−	+	+	−		**O3b**	SRR5270314	K28
Kp83	Spain	ESBL	ST346	SHV-12	rectal swab	+	−	+	+	−		**O3b**		nd
Kp100	Spain	ESBL	ST389	CTX-M-10	urine	+	+	-	+	+	C at position 80	**O3**	SRR5270326	Non typeable
Kp102	Spain	ESBL	ST34	CTX-M-10	respiratory origin	+	−	+	+	−		**O3b**	SRR5270325	K38
Kp103	Spain	ESBL	ST389	CTX-M-10	urine	+	+	−	+	+	C at position 80	**O3**		nd
Kp105	Spain	ESBL	ST16	CTX-M-15	urine	+	−	+	+	−		**O3b**		nd
Kp107	Spain	ESBL	ST16	CTX-M-15	urine	+	−	+	+	−		**O3b**	SRR5270324	K51
Kp116	UAE	CR	ST1798	OXA-48-like	sputum	+	−	+	−	−		**non reactive** ^**b**^	SRR5270323	K58
Kp184	UAE	CR	nd	NDM	blood culture	+	+	−	+	+	C at position 80	**O3**		nd
Kp236	UAE		nd	−	blood culture	+	−	+	+	−		**O3b**		nd
Kp237	UAE		nd	−	blood culture	+	−	+	+	−		**O3b**		nd
Kp245	UAE		nd	−	blood culture	+	−	+	+	−		**O3b**		nd
Kp257	UAE		nd	−	blood culture	+	+	−	+	+	C at position 80	**O3**		nd
Kp269	Israel	CR, ESBL	nd	nd	blood culture	+	−	+	+	−		**O3b**		nd
Kp292	Italy	CR, ESBL, colR	ST273	KPC-3	na	+	−	+	+	−		**O3b**		nd
Kp345	Italy	CR	ST273	KPC-3	na	+	−	+	+	−		**O3b**		nd
LA-KP28	USA		nd	nd	endotracheal aspirate	+	+	−	+	+	C at position 80	**O3**		nd
LA-KP38	USA	ESBL	nd	nd	endotracheal aspirate	+	+	−	+	+	C at position 80	**O3**		nd
FSJ28	Germany	ESBL	nd	nd	urine	+	−	+	+	−		**O3b**		nd
FSJ29	Germany	ESBL	nd	nd	urine	+	−	+	+	−		**O3b**		nd
FSJ48	Germany	ESBL	nd	nd	urine	+	−	+	+	−		**O3b**		nd

^a^Only ESBL production (ESBL), carbapenem resistance (CR) and colistin resistance (colR) indicated.

^b^Despite a low amount of high-molecular weight polysaccharide detected by silver staining, no reactivity with any of the mAbs tested.

^c^Capsule type (K-type) of whole-genome sequenced isolates based on *in silico* prediction. nd - not determined. na - not available.

Interestingly, the novel type O3b outnumbered (71%) the classical O3 serotype strains (29%). The novel O3b variant was found among samples from different geographical and specimen origin suggesting a broad distribution of this subtype. The PCR reactivity designed to distinguish O3 and O3b isolates, fully matched the phenotypic analysis performed with the mAbs except for one strain (Kp116), which did not react with any of the mAbs tested.

### Survival of O3 and O3b isolates in human serum

**Figure 5:**
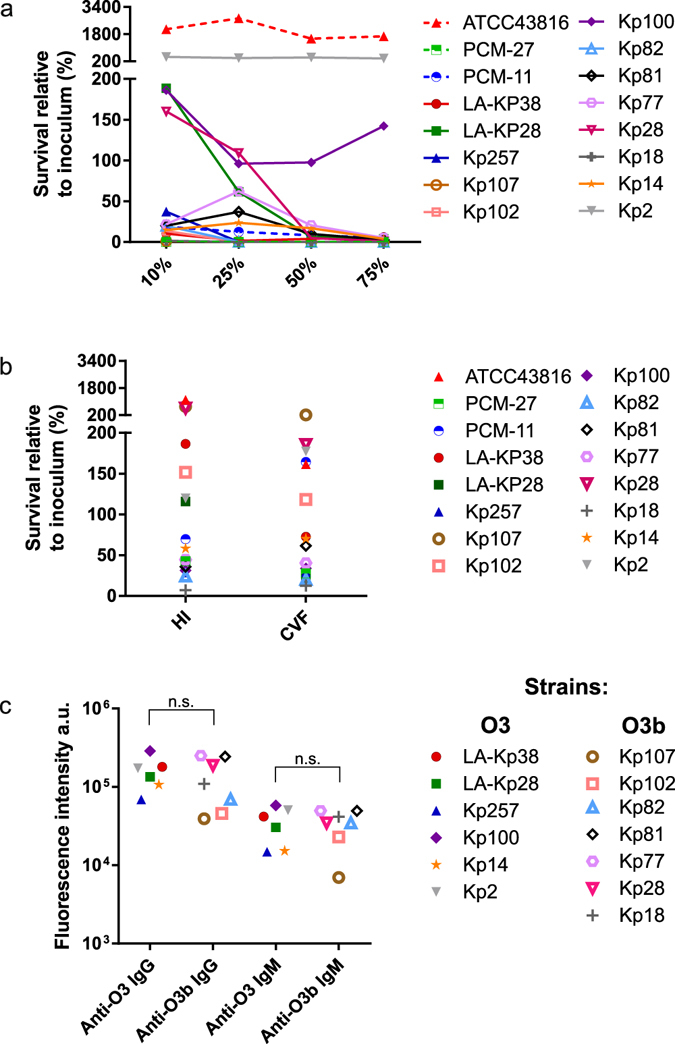
Serum survival of *K. pneumoniae* strains. Strains were incubated in different concentrations of human serum (**a**) or 75% serum complement-inactivated by heat (HI) or cobra venom factor (CVF) treatment (**b**) for 3 h at 37 °C. The recovered bacterial count was expressed as percentage of the initial inoculum. ATCC43816 (O1:K2﻿) and PCM-27 (O2:K27) strains were used as comparators.Graphs show combined results of 2 and 3 experiments for the active and heat inactivated serum experiments, respectively. The same O3 isolates were stained with 10% human serum pool and IgG and IgM antibodies binding to live bacteria were detected with a labelled anti-human IgG or IgM by flow cytometry (**c**). The median fluorescent intensity is shown as arbitrary units. The difference between the staining intensity for O3 and O3b strains was not significant using the Mann Whitney test (p > 0.05; n.s.). O-serotype and capsule type of the strains are indicated in Table 2.

To explore the possibility that the dominance of the O3b subtype strains in our clinical collection originates from higher virulence of this subgroup, we compared survival of six O3, the single O3a and seven O3b strains in a serum pool prepared from 6 healthy individuals (Fig. 5). Importantly, neither an overall association between serum survival pattern and O3 subtype (Fig. 5a) nor a significant difference in pre-existing IgG and IgM titres against O3 versus O3b strains was detected (Fig. 5c).

At higher serum concentrations (50 and 75%) - irrespective of the O3 subtype - all but two strains were killed within 3 hours, confirming susceptibility of the isolates to human serum, particularly when compared to a serum resistant *K. pneumonia* O1:K2 isolate (*K. pneumoniae* ATCC43816). At lower serum concentrations, however, significant differences in the degree of susceptibility were observed; the majority of the strains were highly sensitive even to 10 and 25% human serum, while other strains (e.g. Kp28 and LA-KP28) showed limited growth in 10% human serum, although their survival rapidly decreased at higher serum concentrations. Interestingly while Kp28 and Kp107 belonged to the same O-antigen and capsule serotype (O3b:K51), they showed marked difference in susceptibility to low serum concentration; e.g. in 10% human serum 160% versus 1.1% of the initial inoculum survives, respectively. In some instances, significant bactericidal activity of the complement-inactivated serum was observed (Fig. 5b), implying the importance of non-complement serum components targeting these isolates. In accordance with this, the susceptibility pattern of the clinical strains to the serum pool did not correlate with the corresponding antibody titre as measured by flow cytometry (Fig. 5c).

## Discussion

The widespread and increasing drug resistance seen in *K. pneumoniae* calls for the development of novel, non-antibiotic therapeutic approaches, including active and passive immunization. Strains resistant to all classes of currently available antibiotics were recently described that project the arrival of the post-antibiotic era. Molecular targets of active or passive immune strategies are restricted by the bulky capsular polysaccharide masking most surface antigens of Klebsiella. Different classes of LPS O-antigens, however, were validated to be accessible for antibodies^7, 20, 21^ based on bactericidal effector functions^7, 8^. This study also corroborated the exposure of *K. pneumoniae* O-antigens by demonstrating intense surface staining with O3-specific mAbs in the presence of different capsular polysaccharides.

Another important factor that makes *K. pneumoniae* O-antigens attractive targets for antibody-based approaches is their limited variability. A recent *in silico* study analysing over 600 strains showed that 85% of the isolates belonged to one of the three most common O-antigen types, i.e. O1, O2, and O3^5^. These data are in very good agreement with the seroepidemiology results described decades ago in two large independent studies^3, 6^. Importantly, however, while the O1 serogroup appears to be antigenically conserved, several subserotypes of the O2 serogroup were suggested to originate from the non-stoichiometric modification of the galactan backbone with various side groups^22^. One of these O2 subtypes – that was shown recently to be associated with the epidemic KPC-producing ST258 clone^21^ – was described as an antigenically separate serotype^21, 22^.

In this work, we focused on the characterization of variants within the O3 serogroup. We have reported 3 subtypes of the O3 serogroup that differ in the number of mannose residues within the O-antigen RU. The structure of the archetype *K. pneumoniae* O3 RU, which is identical to that of *E. coli* O9 and *Hafnia alvei* PCM 1223, was described decades ago^9^ as a pentamannose structure. Additionally, in the case of *E. coli* O9, a subtype with tetramannose RU was also identified^16^. This represents an antigenically distinct structure based on different reactivity to mAbs^23^ and hence was termed serotype O9a. The O9a variant was suggested to originate from recombination of corresponding *K. pneumoniae* sequences into the *rfb*(*wb*) operon of the O9 archetype strain^24^. Later, a single point mutation (C80R) within *wbdA* encoding the bifunctional mannosyltransferase was proven to be responsible for the switch from the penta- to the tetramannose RU structures^16^. The tetramannose structure corresponding to *E. coli* O9a has not been described in *K. pneumoniae* before. This work could find only one strain with this structure, which is proposed to be termed O3a in order to conciliate the nomenclature with corresponding *E. coli* O-antigens. The O3a strain carried the point mutation described to be essential for the synthesis of the tetramannose RU. Furthermore, this study has introduced an additional novel subtype that expresses tri-mannose RUs designated as the O3b subtype. Besides carrying the point mutation encoding the amino acid substitution C80R, the whole O3b WbdA enzyme showed significant divergence from that of O3. An even higher sequence variability was found within WbdD. The two different variants of these two genes were also described in a recent study performing *in silico* analysis of whole genome sequencing data^5^. Given that WbdA and WbdD form a complex for the concerted elongation and termination of O-antigen chains, these simultaneous mutations are not surprising. The WbdA-D machinery has probably co-evolved to maintain the optimal chain length of the novel O3b type. Based on staining of purified LPS molecules, the overall length (size) of the O3b chains is somewhat shorter than the O3 chains. Given, however, the 40% smaller RU size, the modal distribution, i.e. the average number of RU within the O-antigen chain, appears to be maintained.

The O-antigen of all O3 variants described in this study are terminated by MeP groups. This justifies the conservation of genes encoding the transfer of terminated chains, since the transport machinery is specific for the chain terminator group^18, 25^. It is tempting to speculate that this conserved terminator group constitutes part of the epitope of the cross-reactive mAbs, however, this still awaits to be addressed experimentally.

The analysis of distribution of the different subtypes within *K. pneumoniae* O3 serogroup revealed that the novel O3b type outnumbered the clinical isolates expressing the pentamannose RU structure. The same conclusion was drawn by the authors of the recent *in silico* epidemiology study^5^. The current high prevalence of O3b and the fact that this subtype has not been described earlier (neither among *K. pneumoniae* O3 nor among *E. coli* O9 ser﻿ogroup isolates) suggests a recent emergence. Nevertheless, we could find O3 strains from various clinical samples isolated at three different continents representing a global distribution. The worldwide spread in spite of the proposed recent emergence suggests an evolutionary advantage through the expression of the O3b mannan antigen. The novel O-antigen type may afford higher fitness/virulence or alternatively serve as an immune evasion strategy to overcome pre-existing antibody titres against common mannan structures. In order to address these questions experimentally, we compared serum resistance of a panel of O3 and O3b strains. In general, with a few exceptions, most isolates were serum susceptible at high serum concentrations. At lower serum concentration, however, significant differences in survival were observed. Importantly, there was no clear association between the O-antigen type and the resistant phenotype. Interestingly, two isolates of the same serotype (O3b:K51) showed clear difference in serum resistance suggesting that other factors besides O- and K-antigens may play an important role in serum resistance. To test the second hypothesis, i.e. that newly emerging O-types face lower pre-existing antibody levels and consequently could serve as immune evasion strategy, flow cytometry was performed with a collection of O3 and O3b strains. No significant difference in the amount of specific IgG or IgM titres could be detected by surface staining of O3 and O3b strains^11^. Anyhow, since serum antibody titres against a given strain did not correlate with its survival, serum mediated killing likely does not mainly rely on the classical complement pathway. Further studies are needed to elucidate the importance of different O3 subtypes in the evolution of *K. pneumoniae* virulence.

The phenotypic and genotypic tools used in our epidemiology study (i.e. reactivity to specific mAbs, PCR and sequencing) correlated very well, confirming that the observed sequence divergence can be used as surrogate for the structural differences. Consequently, for sero-epidemiology studies PCR appears to be a reliable tool with the limitation of potentially missing rough strains. A PCR-based O-serotyping has been described to distinguish between the traditionally known 9 serotypes^26^. Complementing this set with primers distinguishing O3b from O3 could further discriminate the different subserotypes.

The mAbs described in this study may have importance beyond diagnostic applications. As described above, passive immunization with a cocktail of O-antigen specific mAbs is envisioned to provide a reasonable coverage against the vast majority of *K. pneumoniae* isolates. Nevertheless, the number of mAbs in the cocktail is a critical factor both in terms of regulatory complexity and production costs. Serogroup O3 is one of the major O-antigen types in Klebsiella, and therefore cannot be excluded from a broad-spectrum approach. The existence of different antigenic subtypes within serogroup O3, however, requires the use of cross-reactive antibodies to cover all subtypes. Here, we provide evidence that mAbs with cross-reactive potential to O3-O3a-O3b strains can be developed. While our results demonstrate that the target O-antigens of live bacteria, irrespective of their O3 or capsular polysaccharide type, are accessible to a cross-reactive antibody, further studies should evaluate the protective efficacy of the antibody in *in vitro* and *in vivo* experimental models.

## Materials and Methods

### Bacterial strains

PCM-11 was obtained from the Polish Collection of Microorganisms (PCM). A non-encapsulated mutant of a prototype O3:K55 strain 5505 (5505Δ*cps*) was kindly provided by S. Kaluzewski (National Institute of Public Health - National Institute of Hygiene, Warsaw, Poland). Clinical isolates were provided by S. Melegh (Hungary), M. Gniadkowski (Poland), M. Assous (Israel), J. Hudcova (USA), F. J. Schmitz (Germany), A. Sonnevend (UAE), and A. Valverde (Spain).

Bacteria were routinely grown in LB broth. Clinical isolates were cultured on either chromID® CARBA SMART or chromID® ESBL plates (Biomérieux, France). Non-resistant strains were cultured on chromID® CPS® elite plates or on TSA plates (both from Biomérieux, France). PCM-11 was biochemically characterized using the API 20E kit (Biomérieux, France) and the results were analysed by the APIwebTM software (Biomérieux, France).

Mouse immunizations were performed using strain PCM-11. *K. pneumoniae* strains Kp81, PCM-11, and 5505Δ*cps* (representatives of the different subtypes) were used for biochemical analysis.

### Antibody discovery

6–8 weeks old female BALB/c mice were immunized with a sublethal dose (10^7^ CFU) of live *K. pneumoniae* O3a (PCM-11) intravenously. Following two subsequent booster immunizations each two weeks apart, O3-specific titres were determined from test sera by ELISA using a purified biotinylated O-antigen (deriving from 5505Δ*cps*). Mice showing the highest specific titres received a final boost with formalin-killed whole bacterial cells and 4 days later the splenocytes were fused to generate murine hybridomas at the Monoclonal Antibody Facility of the University of Vienna. Specific clones were selected by testing supernatants of subcultured hybridoma clones in ELISA and flow cytometry. Poly-reactive clones were counter-selected using unrelated *K. pneumoniae* O-antigens (ELISA) and bacterial cells (flow-cytometry). Isotype of the selected mAbs was determined with the Quick Kit for mouse monoclonal isotyping (Sigma), showing that mAbs 1G6 and 2F8 belong to IgG1 and IgG3 isotypes, respectively. Animal experiments were reviewed and approved by the Arsanis Animal Welfare and Ethics Committee and performed according to Austrian Law (BGBl. I Nr. 114/2012) as approved by the respective competent authority (Magistratsabteilung 58, Vienna, Austria) under the animal testing permit (M58/005326/2012/5).

### PCR and primers

The primers used to identify the different O3 subtypes are shown in Table 3. Template DNA was purified from o/n bacterial cultures with the DNeasy blood & tissue kit (Qiagen). GoTaq® Green Master Mix (Promega) was used to amplify fragments in an Eppendorf Mastercycler Pro Thermal Cycler. Amplicons were visualized in a 1% agarose (Fisher Scientific) gel containing 0.8% GelRed™ Nucleic Acid stain (Biotum). The *wbdA* amplicon of O3/O3a strains was purified from agarose gel using the QIAquick Gel Extraction Kit (Qiagen) and was Sanger sequenced with the wbdA fw and rev primers at Microsynth Austria GmbH. Sequencing results were analysed with CLC Main Workbench 6.7.1.

**Table 3:** Primers used in the study.

primer	Sequence	Annealing temperature	Fragment size (bp)	Other
wzm fw	5′-GCGATCTATCGCTACCGTGG-3′	60 °C	537	pan-O3 specificity
wzm rev	5′-CTGCAGCAGGATATTGACGAAC-3′			
wbdD O3b	5′-CAGTACTATCTGCTTCGTCAG-3′	56 °C	812	O3b specific
wbdA O3b	5′-GCAAGTTCACGAGCTAGTGTG-3′			
wzt fw	5′-CCATCTAAATGGAACCGGGTC-3′	58 °C	1227	O3 and O3a specific
wzt rev	5′′-CTTAAGATCGATGACACCCCAG-3′			
wbdA fw	5′-GATTGATGTCCAGGGTTACC-3′	58 °C	800	To amplify and sequence *wbdA*
wbdA rev	5′-TCAGGATGCACCTTATACGC-3′			

### LPS preparation

LPS was isolated by the hot phenol/water method and purified by dialysis and ultracentrifugation as previously described^27^. The O-specific polysaccharides (O-PS) and different oligosaccharide components were released by mild acidic hydrolysis (1.5% CH_3_COOH, 20 min., 100 °C) and fractionated as previously described^27^. Obtained poly- and oligosaccharides were fractionated by gel filtration on Bio-Gel P-10 (-400 mesh) (Biorad, USA) or HW-40F (Tosoh Bioscience LLC, USA) yielding fractions 1a, 1b, 1c, 2, 3 and 4, as previously described^27^. For immunoblotting, small scale LPS preparations were performed using a commercial kit (Intron).

### O-PS structural analysis

Methylation and sugar analyses of O-PS (1a fractions) were performed as described earlier^27^ according to the method described by Ciucanu and Kerek^28^. NMR spectra of O-PS (1a fractions) were obtained using an Avance III 600 MHz (Bruker BioSpin, Germany) spectrometer equipped with a 5 mm QCI cryoprobe with z-gradients. NMR spectra of isolated O-PSs were obtained for D_2_O solutions at 25 °C using acetone as an internal reference (δ_H_/δ_C_ 2.225/31.05 ppm), processed and analyzed as described earlier^27^. Briefly, signals were assigned based on 1D ^1^H NMR spectra and 2D experiments COSY, clean-TOCSY, NOESY, HMBC, HSQC-DEPT, and HSQC-TOCSY. The excitation sculpting pulse sequence was used for suppression of water resonances. The mixing times in clean-TOCSY experiments were 30, 60, and 100 ms. The delay time in HMBC was 60 ms, and the mixing time for NOESY was 200 ms. For P-H correlation between MeP and O-PS, ^31^P, ^1^H HMQC and HMQC-TOCSY experiments with *J*_P,H_ of 8–11 Hz were used. The processed spectra were assigned with the use of SPARKY. Negative ion mode MALDI-TOF MS of O-PS was carried out on a Bruker Reflex III time-of-flight (TOF) instrument. 2,5-Dihydroxybenzoic acid (10 mg/ml, acetonitrile/0.2 M citric acid, 1:1) was used as a matrix. O-PS (1 mg/ml) was mixed with the matrix solution in ratio 1:1 to prepare O-PS solutions (1 mg/ml). External calibration in the negative-ion mode was applied using the Peptide Calibration Standard II (Bruker Daltonics, Germany). Ions were interpreted with the use of GlycoWorkbench software^29^.

### O-PS biotinylation

*K. pneumoniae* 5505Δ*cps* O-PS was biotinylated using the carboxyl group of Kdo residue. Briefly, O-PS (10 mg) was dissolved in 1 ml of 0.2 M borate buffer (0.2 M, pH 9.0), and 13.5 mg of EZ-Link amine-PEGn-Biotin (Thermo Scientific, USA) was added to the solution (50:1 molar ratio of PEG-biotin to O-PS) and incubated 1 h at 37 °C. Then NaBH_3_CH solution (160–200 μl of ALD Coupling Solution, Sterogene, USA) was added at day 1, 2, 3, 4 of incubation. Biotinylated O-PS was purified by HPLC on TSKgel® G3000PW column (Tosoh Bioscience GmbH, Germany) using mQ water as the eluent. Biotinylation level of the O-PS was measured by HABA/Avidin reagent (SIGMA, USA) according to manufacturer instructions. The yield of biotinylation was 6 mg of 20%-biotinylated O-PS.

### LPS staining and immunoblots

One μg of LPS was separated by SDS-PAGE and stained with ProQ® Emerald 300 lipopolysaccharide staining kit (LifeTechnologies). Alternatively, separated LPS samples were transferred to PVDF membranes for immunoblotting. Membranes were reacted with 1 µg/ml of murine mAbs and secondary HRP-labelled goat anti-mouse IgG at a dilution of 1:40,000 (Southern Biotech). Blots were developed by ECL Prime solution reagent (GE Healthcare).

### Flow cytometry

Bacteria at mid-log phase of growth were washed and reacted (2 × 10^6^ CFU per reaction) with O3 specific mouse mAbs (40 µg/ml) or with 10% human serum pool in HBSS buffer with 0.5% BSA followed by staining with 4 μg/ml of Alexa Fluor® 488-conjugated goat anti-mouse IgG, anti-human IgG or anti-human IgM secondary antibodies (Thermo Scientific). Bacteria were counterstained with 5 μM SYTO-62 nucleic acid stain (Thermo Scientific) and measured in a CytoFlex Flow Cytometer (Beckman Coulter). Data were analysed using the FCS Express software version 5 (De Novo Software).

### Serum survival

Serum samples from Blutzentrale Linz and Vienna (Austria) were collected from 6 volunteers and equal volumes of the individual sera were pooled to generate a normal human serum pool. Serum samples were kept frozen at −80 °C.

Complement was inactivated either by incubating the serum at 56 °C for 60 min (heat inactivated) or by incubating with 2 U/ml Cobra Venom Factor (Quidel) at 37 °C for 60 minutes. In the serum survival assay, 3–5 × 10^4^ CFU/mL of mid-log phase bacteria were incubated at 37 °C in 10, 25, 50 or 75% serum and plated at 3 h time-point. The recovered CFU was correlated to the initial inoculum as determined by colony counting after plating.

### Genome sequencing

Mid-log (OD_600nm_ ~0.5) cultures of bacteria were harvested and DNA was extracted using Genomic-tip 100/G and Genomic DNA buffer set (Qiagen) according to the manufacturer’s instruction specific to bacteria. The quality of the genomic DNA was assessed using the Fragment Analyzer (AATI, USA). Samples passing quality control were further processed using automatic library preparation method (GATC Biotech) and sequenced on a HiSeq. 2500 (Illumina) with 50 bp or 100 bp paired-end mode. On average 18 million read pairs and an average coverage of 341x was achieved. The sequence reads were assembled using CLC bio (Qiagen) into long contiguous sequences. The genes encompassing the *rfb* operon – *manC, manB, wzm, wzt, wbdD, wbdA, wbdB, wbdC* were extracted from the archetype O3 strain 636/52 (AB795941.1), translated into the amino acid sequence and used for O3 antigen screening. The homology of the amino acid sequence to the archetype was calculated and used for generating the *heatmap* using the R package hclust. Multiple alignments of WbdD protein sequences were constructed using progressive alignment method where multiple alignments are built through the successive construction of pairwise alignments. Based on pair-wise distances of multiple protein alignments, a phylogenetic tree was constructed using UPGMA (Unweighted Pair Group Method using Arithmetic averages) clustering algorithm. Similar method was applied to generate phylogenetic trees for WdbA.

*In silico* capsular prediction was performed by screening the isolates for 79 various capsular polysaccharide synthesis (*cps*) regions published recently^30^. The whole genome sequence reads from isolates were mapped to all 79 cps types and the respective *cps* region coverage was computed. The result of the *cps* mapping is shown in the Supplementary Table 1. To those isolates which showed coverage >95% of a specific *cps* region, a capsule type (K-type) was assigned (Table 2).

The assembled *rfb* operons from PMC-11 and Kp81 were deposited to NCBI’s GenBank database (Accession IDs: KY652949 and KY652950 respectively). Whole genome sequencing reads from 13 O3 isolates were deposited to NCBI’s sequence read archive (SRA) under the bioproject ID: PRJNA375798 and the samples specific accession numbers are listed in Table 2.

## Electronic supplementary material

Supplementary Information
