# Supplementary material for: Discovery of monoclonal antibodies cross-reactive to novel subserotypes of K. pneumoniae O3
Source: Sci Rep. 2017 Jul 26;7:6635. doi: 10.1038/s41598-017-06682-2 (PMC5529442; doi:10.1038/s41598-017-06682-2)
Supplement: Supplementary file 1 — Supplementary Information [file 41598_2017_6682_MOESM1_ESM.pdf]

Supplementary Information

Development of monoclonal antibodies cross-reactive to novel subserotypes of *K. pneumoniae* O3

Luis M. Guachalla, Katarina Stojkovic, Katharina Hartl, Marta Kaszowska, Yadhu Kumar, Benjamin Wahl, Tobias Paprotka, Eszter Nagy, Jolanta Lukasiewicz, Gabor Nagy, Valéria Szijártó

Supplementary Table 1 Sequence coverage of the *cps* locus of whole-genome sequenced *K. pneumoniae* isolates against 79 different cps regions. Colors indicate the coverage, with increasing coverage from green to red.

CPS type	Kp107	Kp14	Kp100	Kp28	Kp62	Kp18	Kp81	Kp2	Kp102	Kp116	Kp82
K10	41	39	37	39	38	38	36	38	39	38	42
K11	43	38	39	38	39	40	39	38	38	37	41
K12	34	32	42	31	33	32	29	30	32	29	33
K1	45	45	43	43	46	44	43	46	43	61	49
K13	41	41	40	38	41	40	39	41	39	42	46
K14	37	38	46	35	37	36	36	36	35	35	40
K15	48	46	44	47	51	45	43	42	48	36	41
K16	37	38	35	35	38	36	34	35	35	55	41
K17	35	35	47	34	35	33	33	33	34	33	41
K18	30	30	44	30	31	30	28	30	30	28	32
K19	35	30	44	32	31	29	28	31	31	29	33
K20	47	46	43	44	45	43	42	43	43	43	47
K21	41	41	39	39	39	38	38	39	38	38	43
K22	34	34	32	33	33	31	31	30	33	31	36
K2	45	45	43	43	45	44	41	43	44	46	47
K23	41	38	49	38	38	36	35	35	37	34	39
K24	47	47	43	45	52	44	42	44	45	45	47
K25	39	40	38	38	37	37	37	39	39	36	39
K26	25	22	15	19	19	20	19	22	18	18	25
K27	38	37	35	37	37	37	36	35	38	35	38
K28	45	46	43	44	46	43	44	44	44	46	100
K29	29	27	19	21	22	23	20	24	20	18	28
K30	45	46	44	44	45	43	43	45	44	45	48
K31	54	56	51	52	54	52	52	51	51	52	55
K32	23	19	18	15	14	13	13	15	15	12	22
K3	40	42	41	38	41	37	38	39	38	38	42
K33	29	29	27	25	27	26	25	27	25	24	29
K34	39	38	37	38	38	38	36	36	39	36	39

K35	32	79	23	26	76	27	25	26	26	25	32
K36	35	34	32	33	32	32	32	31	32	31	34
K37	34	33	31	33	33	31	31	30	33	31	36
K38	36	32	32	35	33	33	30	31	99	30	31
K39	41	43	42	41	44	42	42	41	42	39	43
K40	25	19	30	23	24	23	22	19	23	15	16
K41	21	18	17	16	14	17	14	16	14	13	17
K42	20	18	16	16	16	16	17	15	16	16	23
K4	33	31	32	31	31	32	31	32	31	33	23
K43	41	42	42	39	42	39	37	41	40	39	44
K44	21	19	26	15	15	17	14	17	16	14	23
K45	32	30	42	29	30	27	27	28	30	27	34
K46	40	41	38	38	41	39	38	39	40	39	43
K47	35	32	47	32	34	30	29	31	32	29	34
K48	31	30	45	27	31	31	28	31	28	29	33
K49	41	41	42	39	42	39	38	42	39	39	44
K50	65	60	56	65	59	70	73	48	76	71	64
K51	100	36	35	100	36	35	33	33	39	34	37
K52	36	35	46	35	35	32	32	33	36	32	36
K5	43	42	40	41	41	40	39	41	41	42	46
K53	39	39	49	36	39	36	34	40	36	35	42
K54	41	40	38	40	40	40	38	40	39	62	45
K55	33	32	45	30	31	28	29	29	30	28	35
K56	32	31	42	29	32	29	26	32	29	27	31
K57	52	49	47	49	51	47	47	47	48	44	47
K58	39	38	38	36	38	38	36	40	36	96	43
K59	24	17	15	14	15	16	13	17	16	13	22
K60	41	42	40	40	42	40	39	98	39	40	44
K61	43	44	41	41	43	41	40	41	41	41	52
K62	44	44	45	43	44	43	41	44	44	42	47
K6	28	23	23	26	23	23	22	23	22	23	26
K63	45	43	43	43	45	41	41	43	43	42	47
K64	37	39	47	36	40	36	35	37	36	36	41
K65	28	23	18	20	18	18	18	22	18	17	28
K66	38	33	28	28	29	29	27	30	26	26	37

K67	26	26	31	22	23	23	22	24	22	22	30
K68	20	15	9	12	13	14	11	12	12	12	20
K69	21	17	13	14	15	16	17	15	13	10	17
K70	18	17	18	12	11	15	11	14	11	9	15
K71	42	41	55	40	42	39	39	45	40	39	45
K72	21	18	21	15	15	14	15	15	14	13	23
K7	38	39	36	37	38	36	36	37	37	37	40
K74	30	28	21	21	23	23	21	25	21	20	29
K79	21	18	20	14	15	14	13	14	14	12	21
K80	36	38	47	34	37	33	33	36	34	34	38
K81	28	29	45	28	29	29	27	29	31	26	31
K82	24	19	15	15	18	18	16	15	16	14	25
K8	36	35	32	34	34	33	35	33	33	32	37
K9	39	35	48	35	35	32	32	33	36	31	36
KN1	31	29	45	28	31	30	27	31	28	28	33
KN2	41	37	36	39	37	38	35	34	40	34	37

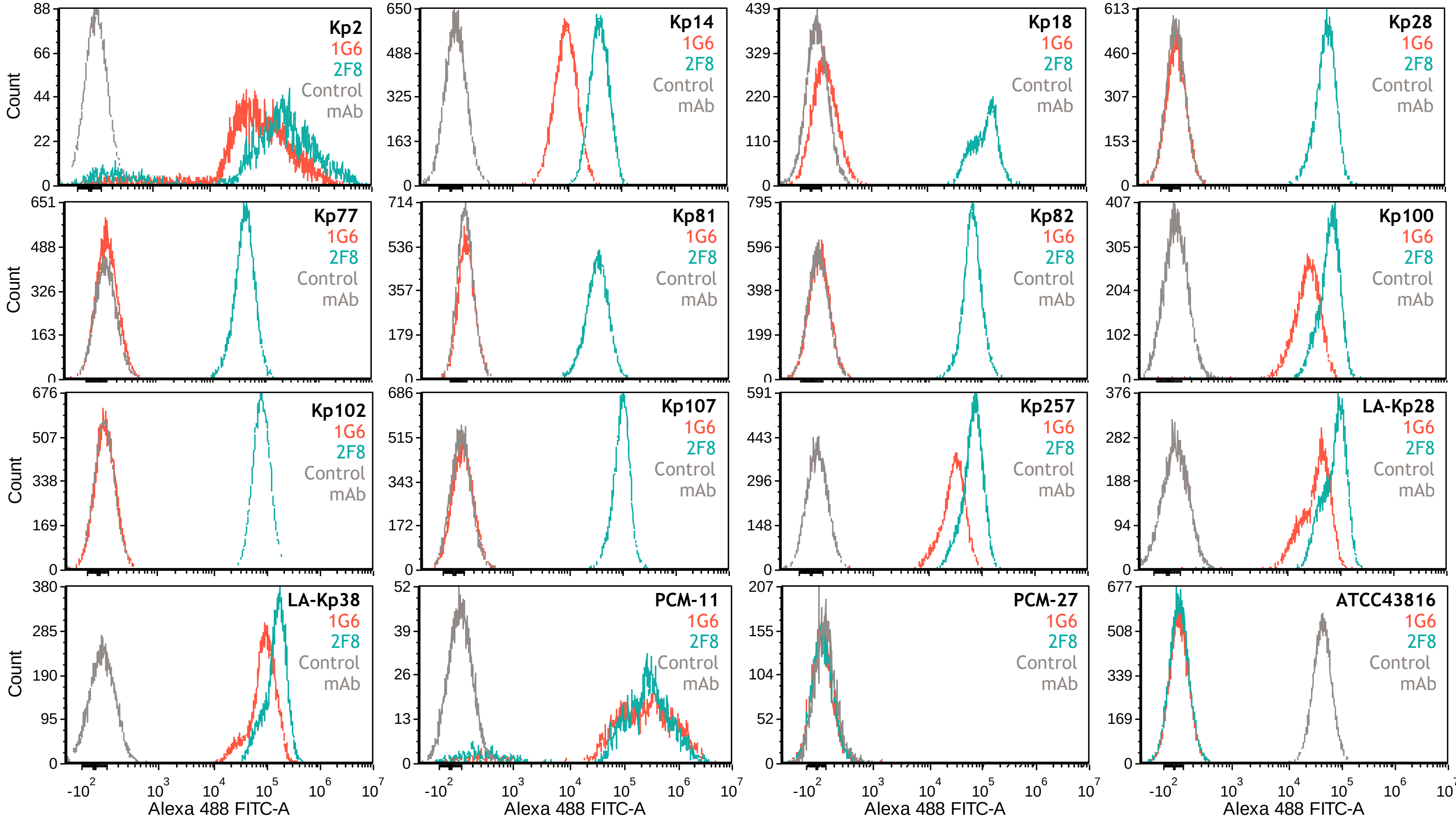

Supplementary Figure 1. Surface staining of clinical *K. pneumoniae* isolates. Histograms show fluorescence intensity of the surface stained bacteria reacted with 40 µg/mL of the respective test antibody. A specific anti- O1 (D-Gal-II) antibody was used as a control mAb. Strains PCM-27 (O2a) and ATCC43817 (O1:K2) were used as control isolates.

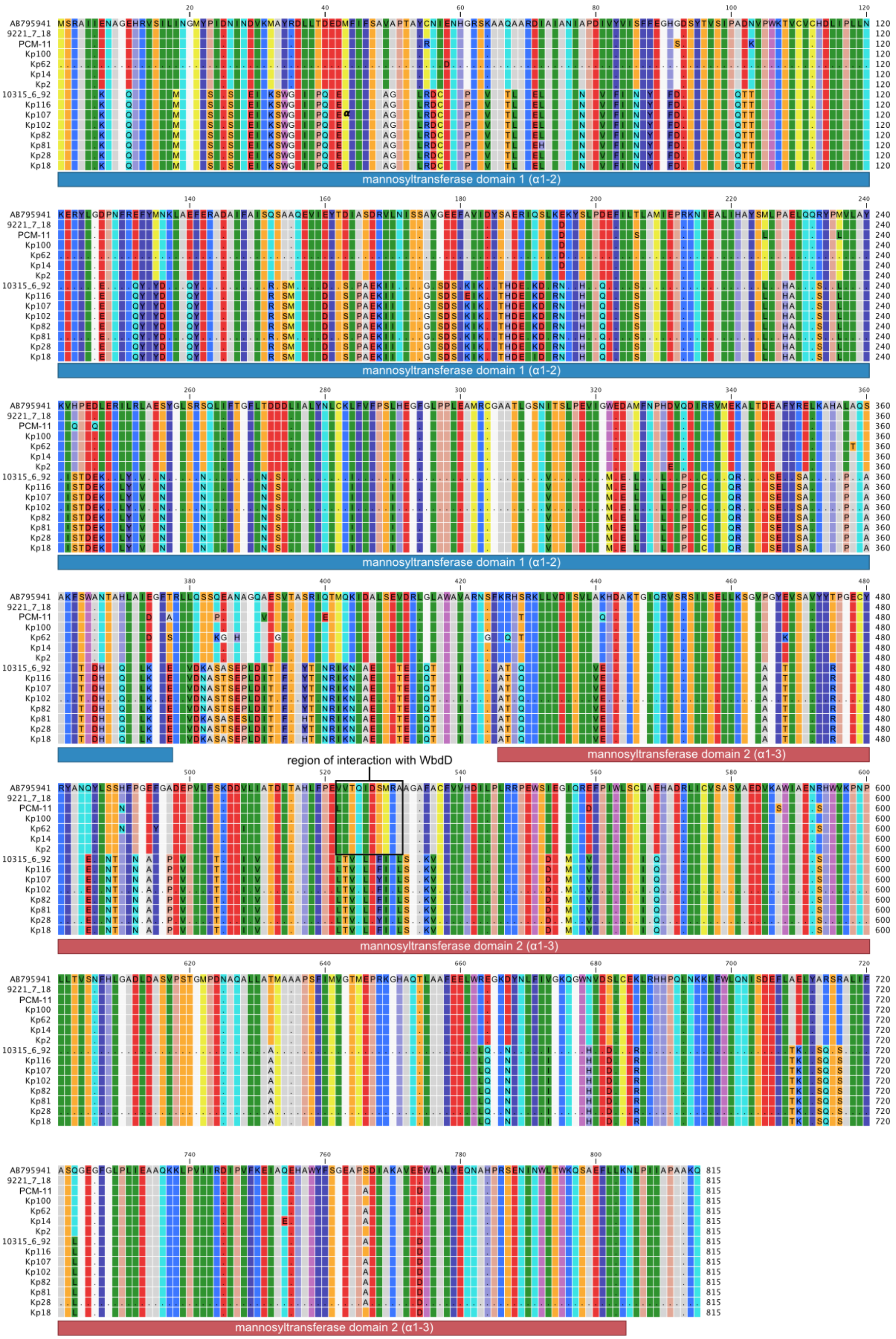

Supplementary Figure 2. Alignment of WbdA from different O3 subtype strains. Alignment of individual WbdA proteins of archetype O3 strain 636/52 (AB795941.1), PCM-11 (O3a) and 11 clinical O3 strains (4 belonging to the traditional O3, and 7 to the O3b subtype). In addition, the recently described two genotypes “O3 short” (10315_6_92) and “O3 long” (9221_7_18) of O3 *rfb* operons[5] are shown. Enzymatic domains and region of interaction are indicated below the sequences.

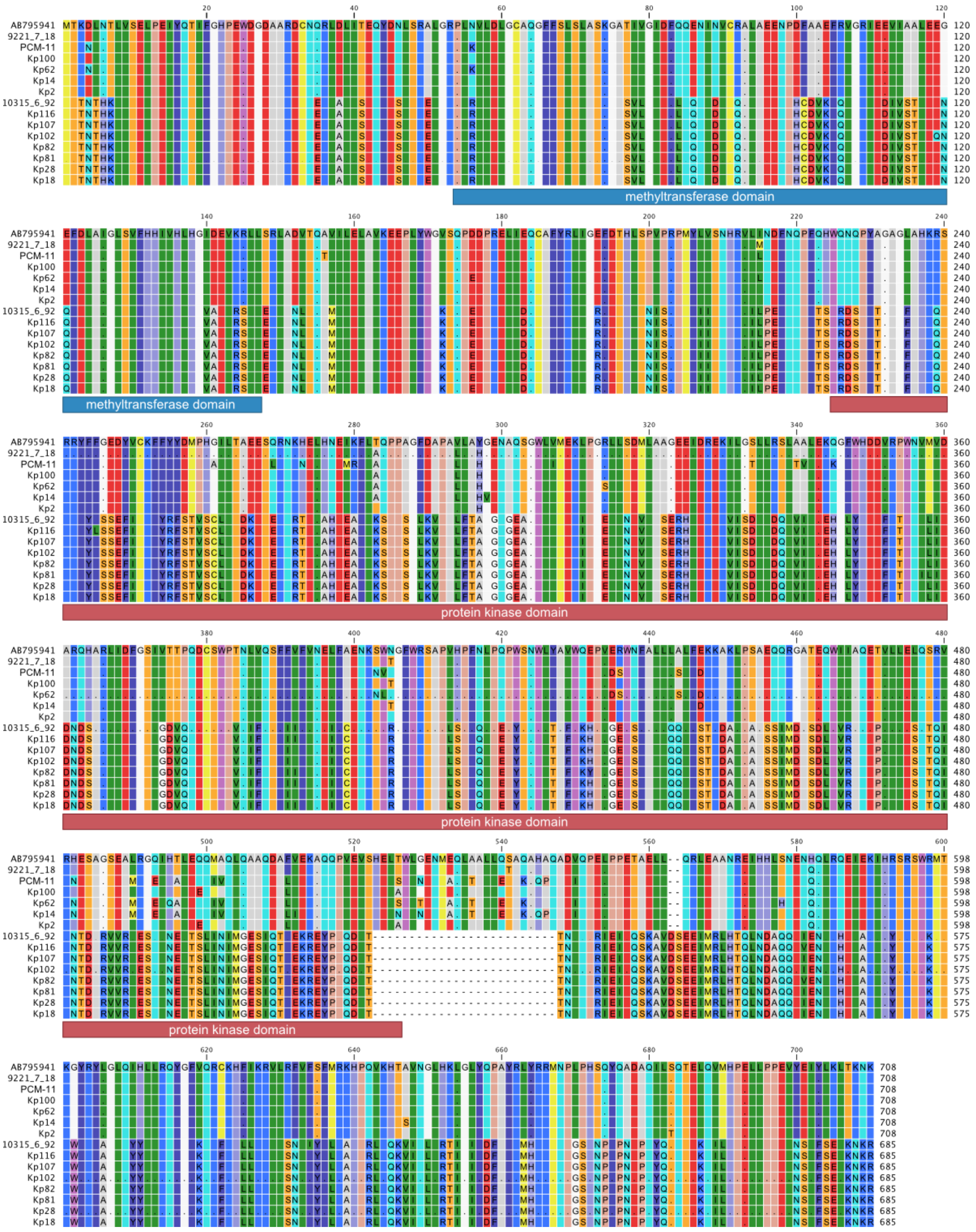

Supplementary Figure3. Alignment of WbdD from different O3 subtype strains. Alignment of individual WbdD proteins of archetype O3 strain 636/52 (AB795941.1), PCM-11 (O3a) and 11 clinical O3 strains (4 belonging to the traditional O3, and 7 to the O3b subtype). In addition, the recently described two genotypes “O3 short” (10315_6#92) and “O3 long” (9221_7#18) of O3 *rfb* operons[5] are shown. Methyltransferase and protein kinase domains are shown below the sequences.
